# RETRACTION: Expression of Factor X in BHK‐21 Cells Promotes Low Pathogenic Influenza Viruses Replication

**DOI:** 10.1155/av/9813437

**Published:** 2025-12-04

**Authors:** Advances in Virology

RETRACTION: S. Shahsavandi, M. M. Ebrahimi, S. Masoudi, and H. Izadi, “Expression of Factor X in BHK‐21 Cells Promotes Low Pathogenic Influenza Viruses Replication,” *Advances in Virology*, no. 2015 (2015), https://doi.org/10.1155/2015/675921.

The above article, published online on 31 December 2015 in Wiley Online Library (https://www.wileyonlinelibrary.com), has been retracted following the agreement of the journal’s Academic Editor, Priyank Purohit; and John Wiley & Sons Ltd.

The retraction has been agreed following an investigation of the concerns raised by *Actinopolyspora biskrensis* on PubPeer [1], which identified several concerns related to Figure 3.

More specifically, the images of BHK‐21/FX and BHK‐21/trypsin cells at 24 and 72‐hour marks contain overlapping features, despite representing different experimental conditions.

As a result of the investigation, the data and conclusions of this article are considered unreliable.

The authors disagree with this retraction.
